# Synthesis, antioxidant and antimicrobial properties of novel pyridyl-carbonyl thiazoles as dendrodoine analogs

**DOI:** 10.3906/kim-2008-8

**Published:** 2020-12-16

**Authors:** Zafer ŞAHİN, Sevde Nur BİLTEKİN, Leyla YURTTAŞ, Şeref DEMİRAYAK

**Affiliations:** 1 Department of Pharmaceutical Chemistry, School of Pharmacy, İstanbul Medipol University, İstanbul Turkey; 2 Department of Pharmaceutical Microbiology, School of Pharmacy, İstanbul Medipol University, İstanbul Turkey; 3 Department of Pharmaceutical Chemistry, Faculty of Pharmacy, Anadolu University, Eskişehir Turkey

**Keywords:** Antioxidant, antimicrobial, thiazole, dendrodoine

## Abstract

Marine compound dendrodoine was first obtained from tunicate species ( *Dendrodo grossularia* ). It has a five-membered ring, namely, it is a heterocycle thiadiazole, which is found rarely in natural sources *.* Following its biological activities, novel analogs have been investigated recently. Synthesis of the analogs for this study is realized with uncommon thiazole closure, including methylene-carbonyl condensation. Structures are elucidated by NMR ( ^1^ , ^13^ C) and HRMS spectrums. As an alkaloid derivative, antioxidant properties were evaluated with DPPH and FRAP assays and antimicrobial effect with microdilution method. Among the series, **3bc-3cf** showed higher antioxidant activity than those having 3 or 4-pyridyl substituents. There is lesser activity for 2-pyridyl activity for 2-pyridyl containing group, which may be a result of intramolecular interactions. No activity was observed against gram-negative bacteria at 250 μg/mL. **3ae** and **3ce** showed activity at 64 μg/mL against *S. aureus* and **3ae** showed activity at 16 μg/mL against *S. epidermidis* gram-positive bacteria. Chloramphenicol showed activity against all microorganisms at 8–16 μg/mL. Sixteen original dendrodoine analogs have been defined by close/higher activity compared to dendrodoine analogs and Trolox.

## 1. Introduction

Marine tunicates are animals living in different deepness levels of oceans and are usually attached to docks. Dendrodoine is an alkaloid that was isolated from *Dendroda grossular* indigenous in North Britain in 1980 [1]. Dendrodoine synthesis was accomplished 4 years after the first isolation. It has a thiadiazole ring which is rarely found in natural compounds, and it is known to be a part of functional synthetic compounds [2]. Beyond its unique thiadiazole ring, it is also a bioisoster of aminothiazoles. Aminothiazoles have a diverse biological spectrum including anticancer, antimicrobial, antioxidant, antidiabetic, anticonvulsant, antiinflammatory, antihypertensive, and protective activities. There are approved drugs, such as meloxicam, cefdinir, famotidine, bearing this moiety [3–5].

Atoms with lone-pair electrons can be very dangerous if they are converted to reactive oxygen species. These reactive molecules can interact with cellular mechanisms. In biological mechanisms, O _2_ reduces to H _2_ O via electron transfer pathways such as NADP/NADPH. And this process produces free-radicals (FRs) or reactive oxygen species (ROSs). [6]. FRs or ROSs have homeostatic functions at normal conditions and they are normally produced in small amounts; however, overexpression of them may propagate harmful chain reactions using DNA, RNA, proteins, and lipids as substrate. Finally, these reactions may be the cause of various pathogenic processes such as tumor growth and oxidative stress [7–9].

**Figure 1:**
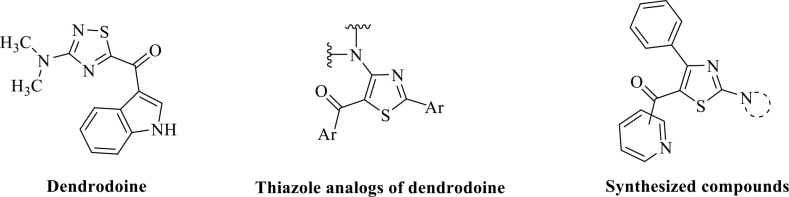
Structures of the dendrodoine analogs.

An analog synthesis is a functional approach in medicinal chemistry. In this respect, natural compounds have been extensively researched. There is only a few studies about dendrodoine analogs (Figure 1), and these studies are usually based on antioxidant activity [1,10-14].

In this study, we have synthesized trisubstituted aminothiazole compounds bearing pyridoyl and phenyl groups and have evaluated their antioxidant and antimicrobial properties. DPPH free-radical scavenging and FRAP iron reducing properties were measured for antioxidant capacity. Besides, antimicrobial properties were evaluated against 8 gram-positive and gram-negative bacteria.

## 2. Materials and methods

### 2.1. Synthesis

The reactants necessary for the synthesis process were purchased from Sigma-Aldrich Chemical Corp. Melting point of title molecules were accomplished by a Stuart melting point apparatus and experiments were performed in duplicate. ^1^ -NMR and ^13^ C-NMR spectrums were recorded in Bruker 300 MHz UltraShield NMR and Bruker 75 MHz UltraShield NMR, respectively. DMSO- *d* *6* was used as solvent and TMS was used as standard. High resolution mass spectrums were recorded in Shimadzu 8040 LC/MS/MS ITTOF system with the electron spray method (ESI). IC _50_ values of the title molecules were calculated using GraphPad Prism software version 7.02.

#### 2.1.1. Synthesis of benzoyl thioureas (2a-f)

Ammonium thiocyanate (500 mmol) was dissolved in acetone. After it becomes a clear solution, 500 mmol benzoyl chloride was added to it quite slowly. The mixture became white blurry after 30 min. An equivalent mole of corresponding amine (500 mmol) was added. The mixture was then boiled for 6 h. The finalization of the process was decided with thin-layer chromatography using ethyl acetate 1:1 petroleum ether solvent system. Precipitation was filtered off, and mixed with water. The obtained solid material was recrystallized using ethyl alcohol [15].

#### 2.1.2. Synthesis of 2-bromoacetylpyridines (1a-c)

Acetyl pyridine (250 mmol) was dissolved in chloroform. After 15 min stirring, 250 mmol bromine (diluted in chloroform) was added quite slowly to the mixture. It was checked with thin-layer chromatography that reaction ended, and then solid material was gathered on the bottom. Because of the basic nitrogen, each compound was formed as HBr salt. Compounds were used as HBr salt in the next step [16].

#### 2.1.3. Synthesis of target compounds (3aa-af, 3ba-bf, 3cc-cf)

**Figure 2:**
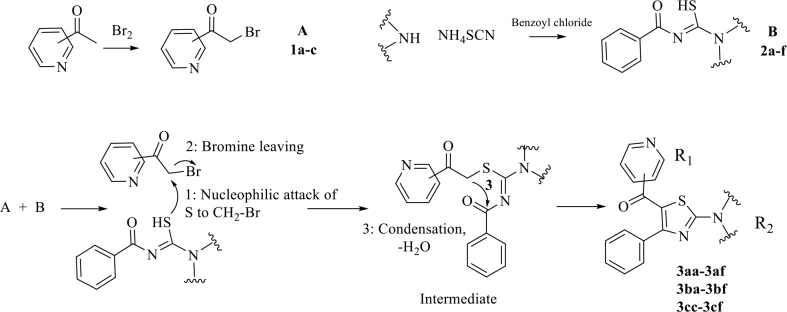
Synthesis of the target compounds.

The target molecules were obtained using a distinct ring closure method including methylene-carbonyl condensation (Figure 2). During reactions, bromoacetylpyridines **(1a-c)** (20 mmol) and **(2a-f)** (20 mmol) were mixed and heated in ethyl alcohol. When it was certain that the reaction ended, the mixture was left for cooling (Table 1). When it reached 15–20 °C, it was poured into cold water (50 mL), and then it was added to water and neutralization was made using NaHCO _3_ . The final compounds were dissolved and recrystallized using ethyl alcohol [17-21].

**Table 1:** R groups of the synthesized compounds.

	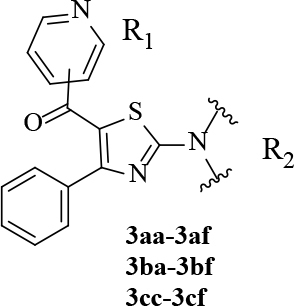						
	R1	R2	Isolated yield (%)		R1	R2	Isolated yield (%)
3aa	2-pyridyle	Dimethylamine	82	3bc	3-pyridyle	Pyrrolidine	83
3ab	2-pyridyle	Diethylamine	75	3bd	3-pyridyle	Piperidine	66
3ac	2-pyridyle	Pyrrolidine	70	3be	3-pyridyle	Hexamethylamine	70
3ad	2-pyridyle	Piperidine	68	3bf	3-pyridyle	Morpholine	84
3ae	2-pyridyle	Hexamethylamine	85	3cc	4-pyridyle	Pyrrolidine	76
3af	2-pyridyle	Morpholine	90	3cd	4-pyridyle	Piperidine	70
3ba	3-pyridyle	Dimethylamine	68	3ce	4-pyridyle	Hexamethylamine	78
3bb	3-pyridyle	Diethylamine	62	3cf	4-pyridyle	Morpholine	69

**(2-(Dimethylamino)-4-phenylthiazol-5-yl)(pyridin-2-yl)methanone (3aa)**

Yield: 82%. m.p.: 160–161 °C. ^1^ -NMR (300 MHz, DMSO-d6, ppm) δ: 3.19 (6H, s, N(CH _3_ ) _2_ ), 7.25–7.32 (3H, m, Ar-H), 7.43–7.53 (3H, m, Ar-H), 7.83 ( ^1^ , d, *J* : 7.76 Hz, Ar-H), 7.91 ( ^1^ , dt, *J* : 8.58, 1.70 Hz, Ar-H), 8.52 ( ^1^ , d, *J* : 4.92 Hz, Ar-H). ^13^ C-NMR (75 MHz, DMSO-d6, ppm) δ: 40.5(N-CH _3_ ), 116.3, 123.2, 126.8, 127.8, 128.8, 129.9, 136.9, 137.9, 148.2, 154.9 (thiazole C _4_ ), 163.8 (pyridine C2), 173.9 (thiazole C2), 182.7 (C=O). HRMS (m/z): [M+H] ^+^ calcd for C _17_ H _15_ N _3_ OS: 310.1005; found: 310.1009.

**(2-(Diethylamino)-4-phenylthiazol-5-yl)(pyridin-2-yl)methanone (3ab)**

Yield: 75%. m.p.: 112–114 °C. ^1^ -NMR (300 MHz, DMSO-d6, ppm) δ: 1.22 (6H, t, *J* : 7.03 Hz, (CH _3_ )2), 3.57 (4H, q, *J* : 7.06 Hz, N(CH _2_ )2), 7.23–7.30 (3H, m, Ar-H), 7.42–7.49 (3H, m, Ar-H), 7.80–7.91 (2H, m, Ar-H), 8.48 ( ^1^ , d, *J* : 4.76 Hz, Ar-H). ^13^ C-NMR (75 MHz, DMSO-d6, ppm) δ: 12.8 (CH _3_ ), 48.8 (N-CH _2_ ), 115.9, 123.3, 126.7, 127.3, 127.8, 128.8, 129.6, 129.9, 130.3, 130.8, 136.9, 137.8, 148.2, 155.1 (thiazole C _4_ ), 163.7 (pyridine C2), 172.3 (thiazole C2), 182.9 (C=O). HRMS (m/z): [M+H] ^+^ calcd for C _19_ H _19_ N _3_ OS: 338.1314; found: 338.1322.

**(4-Phenyl-2-(pyrrolidin-1-yl)thiazol-5-yl)(pyridin-2-yl)methanone (3ac)**

Yield: 70%. m.p.: 180-182 °C. ^1^ -NMR (300 MHz, DMSO-d6, ppm) δ: 2.01 (4H, brs, (CH _2_ ) _2_ ), 3.51 (4H, brs, N(CH _2_ ) _2_ ), 7.27–7.32 (3H, m, Ar-H), 7.42–7.53 (3H, m, Ar-H), 7.81–7.91 (2H, m, Ar-H), 8.53 ( ^1^ , d, *J* : 4.66 Hz, Ar-H). ^13^ C-NMR (75 MHz, DMSO-d6, ppm) δ: 25.6 (CH _2_ ), 49.9 (N-CH _2_ ), 115.7, 123.2, 126.8, 127.8, 128.3, 128.7, 129.9, 137.1, 148.2, 155.0 (thiazole C _4_ ), 164.1 (pyridine C2), 170.5 (thiazole C2), 182.5 (C=O). HRMS (m/z): [M+H] ^+^ calcd for C _19_ H _17_ N _3_ OS: 336.1152; found: 336.1165.

**(4-Phenyl-2-(piperidin-1-yl)thiazol-5-yl)(pyridin-2-yl)methanone (3ad)**

Yield: 68%. m.p.: 124.5 °C. ^1^ -NMR (300 MHz, DMSO-d6, ppm) δ: 1.63 (6H, brs, (CH _2_ )3), 3.60 (4H, brs, N(CH _2_ )2), 7.24–7.31 (3H, m, Ar-H), 7.44 (2H, d, *J* : 7.65 Hz, Ar-H), 7.47–7.52 ( ^1^ , m, Ar-H), 7.83 ( ^1^ , d, *J* : 7.58 Hz, Ar-H), 7.90 ( ^1^ , dt, *J* : 7.55, 1.67 Hz, Ar-H), 8.51 ( ^1^ , d, *J* : 4.81 Hz, Ar-H). ^13^ C-NMR (75 MHz, DMSO-d6, ppm) δ: 23.9 (CH _2_ ), 25.3 (CH _2_ ), 49.2 (N-CH _2_ ), 115.9, 123.3, 126.8, 127.8, 128.8, 129.9, 136.9, 137.9, 148.2, 154.9 (thiazole C _4_ ), 163.7 (pyridine C2), 173.6 (thiazole C2), 182.8 (C=O). HRMS (m/z): [M+H] ^+^ calcd for C _20_ H _19_ N _3_ OS: 350.1322; found: 350.1328.

**(2-(Azepan-1-yl)-4-phenylthiazol-5-yl)(pyridin-2-yl)methanone (3ae)**

Yield: 85%. m.p.: 127–129 °C. ^1^ -NMR (300 MHz, DMSO-d6, ppm) δ: 1.55 (4H, brs, (CH _2_ )2), 1.79 (4H, brs, (CH _2_ )2), 3.66 (4H, brs, N(CH _2_ )2), 7.22-7.33 (3H, m, Ar-H), 7.42–7.50 (3H, m, Ar-H), 7.82 ( ^1^ , d, *J* : 7.88 Hz, Ar-H), 7.89 ( ^1^ , dt, *J* : 7.50, 1.73 Hz, Ar-H), 8.49 ( ^1^ , d, *J* : 4.81 Hz, Ar-H). ^13^ C-NMR (75 MHz, DMSO-d6, ppm) δ: 27.5 (CH _2_ ), 115.8, 123.2, 126.7, 127.8, 128.8, 129.9, 136.9, 137.8, 148.2, 155.1 (thiazole C _4_ ), 163.7 (pyridine C2), 173.1 (thiazole C2), 182.7 (C=O). HRMS (m/z): [M+H] ^+^ calcd for C _21_ H _21_ N _3_ OS: 364.1480; found: 364.1478.

**(2-Morpholino-4-phenylthiazol-5-yl)(pyridin-2-yl)methanone (3af)**

Yield: 90%. m.p.: 149–151 °C. ^1^ -NMR (300 MHz, DMSO-d6, ppm) δ: 3.60 (4H, t, *J:* 4.88 Hz, N(CH _2_ )2), 3.74 (4H, t, *J:* 4.36 Hz, O(CH _2_ )2), 7.25–7.33 (3H, m, Ar-H), 7.44 (2H, dd, *J:* 7.49, 1.92 Hz, Ar-H), 7.50–7.55 ( ^1^ , m, Ar), 7.86 ( ^1^ , td, *J:* 7.67, 1.22 Hz, Ar-H), 7.93 ( ^1^ , td, *J:* 7.84, 1.74 Hz, Ar-H), 8.53 ( ^1^ , d, *J:* 4.53 Hz, Ar-H). ^13^ C-NMR (75 MHz, DMSO-d6, ppm) δ: 48.1 (N-CH _2_ ), 65.8 (CH _2_ -O), 116.2, 123.3, 127.01, 127.8, 128.9, 129.9, 136.7, 138.0, 148.2, 154.6 (thiazole C _4_ ), 163.2 (pyridine C2), 174.1 (thiazole C2), 182.9 (C=O). HRMS (m/z): [M+H] ^+^ calcd for C _19_ H _17_ N _3_ O _2_ S: 352.1106; found: 352.1114.

**(2-(Dimethylamino)-4-phenylthiazol-5-yl)(pyridin-3-yl)methanone (3ba)**

Yield: 68%. m.p. : 174–176 °C. ^1^ -NMR (300 MHz, DMSO-d6, ppm) δ: 3.19 (6H, s, N(CH _3_ )2), 7.05–7.17 (4H, m, Ar-H), 7.23–7.26 (2H, m, Ar-H), 7.83 ( ^1^ , td, *J* : 7.82, 2.02 Hz, Ar-H), 8.38–8.42 ( ^1^ , m, Ar-H), 8.52 ( ^1^ , d, *J* : 4.92 Hz, Ar-H). ^13^ C-NMR (75 MHz, DMSO-d6, ppm) δ: 40.3 (N-CH _3_ ), 122.4, 123.2, 128.0, 128.3, 129.1, 129.2, 130.4, 134.6, 136.2, 149.4, 151.5 (thiazole C _4_ ), 161.0 (pyridine C2), 171.9 (thiazole C2), 185.9 (C=O). HRMS (m/z): [M+H] ^+^ calcd for C _17_ H _15_ N _3_ OS: 310.0998; found: 310.1009.

**(2-(Diethylamino)-4-phenylthiazol-5-yl)(pyridin-3-yl)methanone (3bb)**

Yield: 62%. m.p.: 143–144 °C. ^1^ -NMR (300 MHz, DMSO-d6, ppm) δ: 1.23 (6H, t, J:6.90 Hz, (CH _3_ )2), 3.58 (4H, q, *J* : 7.05 Hz, N(CH _2_ )2), 7.05–7.11 (3H, m, Ar-H), 7.14–7.20 ( ^1^ , m, Ar-H), 7.23–7.26 (2H, m, Ar-H), 7.65 ( ^1^ , td, *J* : 8.06, 1.95 Hz, Ar-H), 8.38–8.42 (2H, m, Ar-H). ^13^ C-NMR (75 MHz, DMSO-d6, ppm) δ: 12.7 (CH _3_ ), 46.0 (N-CH _2_ ), 121.7, 123.1, 128.0, 128.3, 129.0, 129.2, 130.4, 134.7, 135.0, 136.1, 149.4, 151.5 (thiazole C _4_ ), 161.1 (pyridine C2), 170.4 (thiazole C2), 185.8 (C=O). HRMS (m/z): [M+H] ^+^ calcd for C _19_ H _19_ N _3_ OS: 338.1323; found: 338.1322.

**(4-Phenyl-2-(pyrrolidin-1-yl)thiazol-5-yl)(pyridin-3-yl)methanone (3bc)**

Yield: 83%. m.p.: 176–177 °C. ^1^ -NMR (300 MHz, DMSO-d6, ppm) δ: 2.02 (4H, brs, (CH _2_ )2), 3.51 (4H, brs, N(CH _2_ )2), 7.05–7.25 (6H, m, Ar-H), 7.64 ( ^1^ , td, *J* : 7.91, 2.09 Hz, Ar-H), 8.37–8.41 (2H, m, Ar-H). ^13^ C-NMR (75 MHz, DMSO-d6, ppm) δ: 25.7 (CH _2_ ), 50.2 (N-CH _2_ ), 122.1, 123.1, 128.0, 128.3, 129.0, 129.2, 130.4, 134.7, 135.0, 136.1, 149.3, 151.5 (thiazole C _4_ ), 161.3 (pyridine C2), 168.3 (thiazole C2), 185.85 (C=O). HRMS (m/z): [M+H] ^+^ calcd for C _19_ H _17_ N _3_ OS: 336.1151; found: 336.1165.

**(4-Phenyl-2-(piperidin-1-yl)thiazol-5-yl)(pyridin-3-yl)methanone (3bd)**

Yield: 66%. m.p.: 143–145 °C. ^1^ -NMR (300 MHz, DMSO-d6, ppm) δ: 1.64 (6H, brs, (CH _2_ )3), 3.62 (4H, brs, N(CH _2_ )2), 7.04–7.20 (4H, m, Ar-H), 7.22–7.25 (2H, m, Ar-H), 7.66 ( ^1^ , td, *J* : 7.98, 2.07 Hz, Ar-H), 8.38–8.42 (2H, m, Ar-H). ^13^ C-NMR (75 MHz, DMSO-d6, ppm) δ: 23.8 (CH _2_ ), 25.3 (CH _2_ ), 49.4 (N-CH _2_ ), 121.8, 123.2, 128.0, 129.2, 130.3, 134.6, 136.2, 149.4, 151.6 (thiazole C _4_ ), 161.0 (pyridine C2), 171.5 (thiazole C2), 186.0 (C=O). HRMS (m/z): [M+H] ^+^ calcd for C _20_ H _19_ N _3_ OS: 350.1322; found: 350.1320.

**(2-(Azepan-1-yl)-4-phenylthiazol-5-yl)(pyridin-3-yl)methanone (3be)**

Yield: 70%. m.p.: 130–132 °C. ^1^ -NMR (300 MHz, DMSO-d6, ppm) δ: 1.56 (4H, brs, (CH _2_ )2), 1.80 (4H, brs, (CH _2_ )2), 3.66 (4H, brs, N(CH _2_ )2), 7.05–7.17 (4H, m, Ar-H), 7.23-7.25 (2H, m, Ar-H), 7.65 ( ^1^ , td, *J* : 7.84 Hz, 1.78 Hz, Ar-H), 8.37–8.42 (2H, m, Ar-H). ^13^ C-NMR (75 MHz, DMSO-d6, ppm) δ: 27.5 (CH _2_ ), 121.7, 123.1, 128.0, 129.2, 130.4, 134.7, 135.0, 136.1, 149.4, 151.5 (thiazole C _4_ ), 161.1 (pyridine C2), 171.2 (thiazole C2), 185.8 (C=O). HRMS (m/z): [M+H] ^+^ calcd for C _21_ H _21_ N _3_ OS: 364.1480; found: 364.1478.

**(2-Morpholino-4-phenylthiazol-5-yl)(pyridin-3-yl)methanone (3bf)**

Yield: 84%. m.p.: 146–149 °C. ^1^ -NMR (300 MHz, DMSO-d6, ppm) δ: 3.61 (4H, t, *J* : 4.37 Hz, N(CH _2_ )2), 3.74 (4H, t, *J* : 4.37 Hz, O(CH _2_ )2), 7.06–7.18 (4H, m, Ar-H), 7.23–7.26 (2H, m, Ar-H), 7.68 ( ^1^ , td, *J* : 7.98 Hz, 2.06 Hz, Ar-H), 8.39–8.44 (2H, m, Ar-H). ^13^ C-NMR (75 MHz, DMSO-d6, ppm) δ: 48.2 (N-CH _2_ ), 65.8 (CH _2_ -O), 122.4, 123.2, 128.1, 129.3, 130.4, 134.4, 134.8, 136.3, 149.5, 151.7 (thiazole C _4_ ), 160.4 (pyridine C2), 172.0 (thiazole C2), 186.3 (C=O). HRMS (m/z): [M+H] ^+^ calcd for C _19_ H _17_ N _3_ O _2_ S: 352.1113; found: 352.1114.

**(4-Phenyl-2-(pyrrolidin-1-yl)thiazol-5-yl)(pyridin-4-yl)methanone (3cc)**

Yield: 76%. m.p.: 169–171 °C. ^1^ -NMR (300 MHz, DMSO-d6, ppm) δ: 2.03 (4H, brs, (CH _2_ )2), 3.52 (4H, brs, N(CH _2_ )2), 7.04–7.09 (2H, m, Ar-H), 7.15-7.24 (5H, m, Ar-H), 8.29 (2H, d, *J* : 4.47 Hz, Ar-H). ^13^ C-NMR (75 MHz, DMSO-d6, ppm) δ: 25.7 (CH _2_ ), 50.2 (N-CH _2_ ), 122.5, 127.9, 129.4, 130.2, 135.0, 146.1, 149.6 (thiazole C _4_ ), 162.0 (pyridine C2,6), 168.6 (thiazole C2), 186.1 (C=O). HRMS (m/z): [M+H] ^+^ calcd for C _19_ H _17_ N _3_ OS: 336.1155; found: 336.1165.

**(4-Phenyl-2-(piperidin-1-yl)thiazol-5-yl)(pyridin-4-yl)methanone** **(3cd)**

Yield: 70%. m.p.: 135–137 °C. ^1^ -NMR (300 MHz, DMSO-d6, ppm) δ: 1.63 (6H, brs, (CH _2_ )3), 3.61 (4H, brs, N(CH _2_ )2), 7.04–7.09 (2H, m, Ar-H), 7.16–7.24 (5H, m, Ar-H), 8.30 (2H, d, *J* : 4.14 Hz, Ar-H). ^13^ C-NMR (75 MHz, DMSO-d6, ppm) δ: 23.7 (CH _2_ ), 25.3 (CH _2_ ), 49.4 (N-CH _2_ ), 121.4, 122.5, 127.9, 129.4, 130.2, 134.9, 146.0, 149.7 (thiazole C _4_ ), 161.7 (pyridine C2,6), 171.7 (thiazole C2), 186.2 (C=O). HRMS (m/z): [M+H] ^+^ calcd for C _20_ H _19_ N _3_ OS: 350.1322; found: 350.1322.

**(2-(Azepan-1-yl)-4-phenylthiazol-5-yl)(pyridin-4-yl)methanone (3ce)**

Yield: 78%. m.p.: 144–146 °C. ^1^ -NMR (300 MHz, DMSO-d6, ppm) δ: 1.56 (4H, brs, (CH _2_ )2), 1.79 (4H, brs, (CH _2_ )2), 3.60 (4H, brs, N(CH _2_ )2), 7.04–7.09 (2H, m, Ar-H), 7.16–7.24 (5H, m, Ar-H), 8.30 (2H, d, *J* : 4.32 Hz, Ar-H). ^13^ C-NMR (75 MHz, DMSO-d6, ppm) δ: 27.5 (CH _2_ ), 53.2 (N-CH _2_ ), 121.3, 122.5, 127.9, 129.34, 130.2, 135.0, 146.2, 149.6 (thiazole C _4_ ), 161.8 (pyridine C2,6), 171.4 (thiazole C2), 186.0 (C=O). HRMS (m/z): [M+H] ^+^ calcd for C _21_ H _21_ N _3_ OS: 364.1475; found: 364.1478.

**(2-Morpholino-4-phenylthiazol-5-yl)(pyridin-4-yl)methanone (3cf)**

Yield: 69%. m.p.: 171–172 °C. ^1^ -NMR (300 MHz, DMSO-d6, ppm) δ: 3.62 (4H, t, *J* : 4.23 Hz, N(CH _2_ )2), 3.74 (4H, t, *J* : 4.2 Hz, O(CH _2_ )2), 7.05–7.10 (2H, m, Ar-H), 7.17–7.25 (5H, m, Ar-H), 8.32 (2H, d, *J* : 4.05 Hz, Ar-H). ^13^ C-NMR (75 MHz, DMSO-d6, ppm) δ: 48.3 (N-CH _2_ ), 65.8 (CH _2_ -O), 122.0, 122.5, 127.9, 129.5, 130.2, 134.8, 145.9, 149.6 (thiazole C _4_ ), 161.3 (pyridine C2,6), 172.2 (thiazole C2), 186.5 (C=O). HRMS (m/z): [M+H] ^+^ calcd for C _19_ H _17_ N _3_ O _2_ S: 352.1106; found: 352.1114.

### 2.2. Antioxidant activity

#### 2.2.1. DPPH radical scavenging assay

2,2-diphenyl-1-picrylhydrazyl (DDPH) sweep test was used for one type of antioxidant activity as reported by Lu et al. [22]. DDPH has a strong absorbance at 517 nm. DPPH starts to react by existence of a hydrogen donor (free-radical scavenger/antioxidant). The color change depends on the number of electrons captured and is determined with spectrophotometric measurement. Compounds and standard gallic acid were prepared in the concentration from 1.25 µM to 25 µM. These, in six different concentrations (1.25 µM, 2.5 µM, 5 µM, 10 µM, 12.5 µM, 25 µM), were added to 190 µL of simultaneously made solution of DPPH (0.1 mM in methanol). The reaction blend was kept in darkness for 40 min, and then the optical density was measured against the blank at 517 nm. The capability of the compounds of scavenging the DPPH radical was calculated as percent inhibition (%).

Here Ac is the absorbance of the control and At is the absorbance of the compounds; DPPH radical scavenging activity (%) = [(Ac – At)/Ac] × 100. After the concentration calculations, IC _50_ values of each compound were calculated using GraphPad Prism. The results were calculated by comparison with the standard antioxidants, gallic acid and ascorbic acid. The assay was carried out in triplicate [22].

#### 2.2.2. Ferric reducing antioxidant power (FRAP assay)

FRAP test was performed with the method of Benzie and Strain’s (1996) with some modifications [23,24]. To prepare FRAP freshly just before use, 300 mM acetate buffer (pH 3.6), 10 mM TPTZ solution (10 mM TPTZ in 40 mM HCl), and 20 mM FeCl3.6H _2_ O in a 10:1:1 ratio were mixed. Ten-microliter compounds of different concentrations (0.312 μM, 0.625 μM, 1.25 μM, 2.5 μM, 3.75 μM, 5 μM) dissolved in DMSO below 1% were mixed with 230 uL of FRAP reagent and 10 uL dH _2_ O in a 96-well microtiter plate. The plate reading at 593 nm was performed after 8 min using an ELISA plate reader. Trolox was the standard antioxidant molecule. The assay was carried out in triplicate.

### 2.3. Antimicrobial activity

Antimicrobial activity test was applied to various gram-positive and gram-negative bacteria strains including *E. coli* (ATCC 8739), *S. aureus* (ATCC 29213), *B.* *spizizenii* (ATCC 6633), *K. pneumoniae* (Clinical isolate), *P. aeruginosa* (ATCC 9027), *E. faecalis* (ATCC 29212), *S. epidermidis* (ATCC 12228), *VRE* (Clinical isolate). These organisms were inoculated to the midlog phase in Muller Hinton Broth (MHB) at 37 °C. Broth microdilution procedure is a more user-friendly method that enables the testing of multiple antimicrobial agents. The method was carried out by the relevant 2018 CLSI standard. Compounds were dissolved in DMSO below 1% concentration and were added to 96-well plates. These compounds were diluted serially to make ten various concentrations ranging between 0.5 μM and 256 μM. Bacterial inoculum suspensions were primed at 1 × 105 cfu/mL concentrations. Plates were incubated at 37 °C for 24 h. Positive or negative control was set to wells with and without bacteria, respectively. Chloramphenicol and sulfamethoxazole were used as standards. Minimum inhibitory concentration (MIC) was determined by visual inspection after the change in turbidity. Experiments were performed in triplicate [25,26].

## 3. Results

### 3.1. Chemistry

Synthesis of compounds was realized in good yield between 65% and 90%. Structures of compounds were enlightened by spectral data. In summary, methyl groups of compounds 3aa and 3ba (N(CH _3_ )2) were observed at 3.19 ppm with 6H integration. Methyl groups of compound 3ab and 3bb were observed at 1.23 ppm with 6H integration, and J values were 7.03 and 6.90, respectively. Methylene group peaks on the same compounds were observed at 3.58 ppm with 4H integration as a quartet. Aromatic hydrogen count was consistent with the total number on the molecules, and the most shifted hydrogen is the one on the neighborhood of pyridine nitrogen. These hydrogens were observed between 8.29 and 8.53 ppm. In ^13^ C NMR, carbonyl peaks were specifically observed approximately at 180 ppm. Following that, 2nd position of thiazole carbon was observed around 170 ppm. The 2nd position of pyridine and 4th position of thiazole were the following peaks for all compounds in descending order. Methyl or ethyl groups in the neighborhood of amine were recorded at 40 and 52 ppm, respectively. Other aliphatic carbons were recorded at 12 and 27 ppm, which represents methyl and ethyl groups on the amine, but not directly bound to nitrogen, respectively. HRMS results satisfied the molecular weights with high accuracy.

### 3.2. Antioxidant activity

Antioxidant activity was performed by two different common methods namely DPPH radical scavenging and ferric reducing ability (FRAP). DPPH activity was measured as IC _50_ values with mM unit whereas FRAP results are presented as Trolox equivalent antioxidant capacity (TEAC). The higher TEAC result means the higher antioxidant capacity.

In DPPH scavenging activity, 2-pyridyl derivatives **(3aa-af)** showed up to 65 mM IC _50_ value, except 3ad. Remarkably, 3 and 4-pyridyl derivatives showed less than 38 mM IC _50_ , except for 3ba and 3bb. In terms of scavenging activity, standard ascorbic acid exhibited 3 mM. The most active compounds in the series were 3cf and 3bd with 28.10 and 26.02 mM, respectively (Table 2).

**Table 2:** IC _50_ values (μM) for DPPH scavenging ability of the compounds. Antioxidant activity of the compounds (5 mM) as measured with FRAP assay. Data represented as mean ± SE from three individual experiments. (C: Compound, aa: ascorbic acid, ga: gallic acid)

C	IC _50_ (mM)	TEAC	C	IC _50_ (mM)	TEAC
	DPPH	FRAP		DPPH	FRAP
3aa	87.95 ± 2.94	103.66 ± 2.12	3bc	26.13 ± 0.08	123.89 ± 0.45
3ab	150.74 ± 3.99	91.43 ± 0.65	3bd	28.10 ± 0.12	121.17 ± 5.21
3ac	74.37 ± 3.11	93.12 ± 1.42	3be	37.93 ± 2.06	120.67 ± 2.90
3ad	34.10 ± 0.65	102.22 ± 1.47	3bf	31.10 ± 0.90	121.65 ± 0.88
3ae	100.18 ± 3.05	90.34 ± 2.87	3cc	37.76 ± 3.04	124.99 ± 1.62
3af	82.47 ± 1.55	101.56 ± 0.67	3cd	31.13 ± 2.40	107.23 ± 2.89
3ba	66.13 ± 2.36	137.43 ± 0.99	3ce	32.84 ± 2.08	122.12 ± 0.98
3bb	95.91 ± 2.24	123.53 ± 0.38	3cf	26.02 ± 1.55	128.88 ± 2.47
aa	3.21 ± 0.01		ga	0.8 ± 0.04	

In FRAP reducing ability, experiments were performed at 5 μM concentrations. To examine the results, compounds showed a narrow range between 90.34 and 137.43 as TEAC. These results are approximately the quarter of gallic acid results on the same assay. In summary, compounds did not show higher or equal activity to standards (Table 2); however, they still have antioxidant capacity as novel chemicals.

### 3.3. Antimicrobial activity

Final compounds were examined for antibacterial properties on 8 microbial strains (Table 3). The results were presented as MIC (μg/mL) values. According to the results, there is not a considerable activity for gram-negative bacteria *E. coli* , *P. aeruginosa* , and *K. pneumoniae* . Compound 3ae (128 μg/mL) was the only compound that showed more than 256 μg/mL activity against these strains. As for gram-positive bacteria, 3ae, 3af, 3cc, 3cd, 3ce, and 3cf exhibited ≥128 μg/mL activity against different strains. Specifically, 3ae and 3ce showed 64 μg/mL activity against S. aureus. 3ae also exhibited 16 μg/mL activity against S. epidermidis. Chloramphenicol was used as a wide-spectrum standard and sulfamethoxazole was used as sulfonamide standard. Chloramphenicol showed 8–16 μg/mL activity against all bacteria as expected. Considerably, its MIC value is not too far from the results of 3ae. Sulfamethoxazole was not effective against gram-positive bacteria except for S. aureus (64 μg/mL). However, it showed high effect against E. coli (8 μg/mL) and P. aeruginosa (32 μg/mL) gram-negative bacteria.

**Table 3:** Screening for MIC of the compounds using the microdilution method. (The capital S represents strains with its number: S1: E. coli, S2: P. aeruginosa, S3: K. pneumoniae, S4: B. spizizenii, S5: S. aureus, S6: E. faecalis, S7: S. epidermidis, S8: VRE. Ch: Chloramphenicol, Sm: Sulfamethoxazole)

C. no	Microorganisms and minimal inhibitory concentration (μg/mL)							
	Gram-negative bacteria				Gram-positive bacteria			
	S1	S2	S3	S4	S5	S6	S7	S8
3aa	>256	>256	>256	>256	>256	>256	>256	>256
3ab	>256	>256	>256	>256	>256	>256	>256	>256
3ac	>256	>256	>256	>256	>256	>256	>256	>256
3ad	>256	>256	>256	>256	>256	>256	>256	>256
3ae	>128	>256	>256	>256	>64	>256	>16	>128
3af	>256	>256	>256	>256	>128	>256	>128	>256
3ba	>256	>256	>256	>256	>256	>256	>256	>256
3bb	>256	>256	>256	>256	>256	>256	>256	>256
3bc	>256	>256	>256	>256	>256	>256	>256	>256
3bd	>256	>256	>256	>256	>256	>256	>256	>256
3be	>256	>256	>256	>256	>256	>256	>256	>256
3bf	>256	>256	>256	>256	>256	>256	>256	>256
3cc	>256	>256	>256	>256	>256	>256	>128	>256
3cd	>256	>256	>256	>256	>128	>256	>128	>256
3ce	>256	>256	>256	>256	>64	>256	>128	>256
3cf	>256	>256	>256	>256	>256	>128	>128	>256
Ch*	>8	>16	>8	>16	>16	>8	>8	>16
Sm*	>4	>32	>256	>256	>64	>256	>256	>256

## 4. Discussion

Synthesis of sixteen compounds was accomplished. In thiazole synthesis, ring closure is accomplished by methylene-carbonyl condensation. This process runs when there is carbonyl attached to thioamide moiety, unlike in the Hantzsch method. In this way, it is possible to synthesize trisubstituted thiazoles. In this method, sulfur basically attacks alkyl halide (CH _2_ -Br) at the first stage and then bromine leaves the structure, prompting nucleophilic substitution. In the final step, the methylene, which stands between the carbonyl and the sulfur, forms a condensation and water withdrawal with the carbonyl arising from the thioamide moiety (Figure 2) [17–21].

Oceans are rich in many living organisms, including truncates and they have many different biologically active compounds. Antioxidant activity is one of the most important of those activities and compounds IC _50_ values are reported to be between 26.02 and 150.74 mM in DPPH test. 3 and 4-pyridyl derivatives showed higher activity compared to 2-pyridyles. Compounds 3ba and 3bb showed less activity compared to the rest of 3 and 4-pyridyl derivatives (3g-p). This can be interpreted as that the cyclic amine substituents is preferable, not alkyl amines (3ba, 3bb). Standard ascorbic acid was measured as 3.21 mM and this activity seems 9 times of our most active compound. However, in another study, the IC _50_ value of ascorbic acid was reported as 38.78 mM [27]. Trolox equivalent antioxidant capacity for FRAP assay was measured between 90.34 and 137.43, where gallic acid TEAC value was 476.8 and caffeic acid was 272 [28]. Synthesized compounds have antioxidant capacity approximately half of the standard caffeic acid and ¼ of gallic acid. According to antioxidant activity results, compounds have considerable antioxidant capacity but not equal to or higher than standard strong antioxidants such as ascorbic acid, gallic acid, and caffeic acid. DPPH results were measured between 1.25 µM and 25 µM concentrations. Interestingly, dendrodoine analog (Figure 1) was found 16.66 times less active compared to Trolox (measured at 12.28 µM [12]) whereas 3cf (IC _50_ : 26.02 µM) had close or higher activity to Trolox (Reported in the literature: 15–52 µM) [29,30]. In this respect, our compounds seem more active than dendrodoine analogs with respect to antioxidant results and have a close activity to Trolox compared to the literature results. Examining the antimicrobial results, compounds do not have significant antimicrobial activity except for 3ae. As a result, 16 dendrodoine analogs have been defined successfully with their antioxidant and antimicrobial properties.
